# A Convenient Strategy for the Voltammetric Direct Determination of Ce(III) in Environmental Waters Using a Multi-Walled Carbon Nanotubes Modified Screen-Printed Carbon Electrode

**DOI:** 10.3390/molecules29061198

**Published:** 2024-03-07

**Authors:** Malgorzata Grabarczyk, Marzena Fialek, Edyta Wlazlowska

**Affiliations:** Department of Analytical Chemistry, Institute of Chemical Sciences, Faculty of Chemistry, Maria Curie-Sklodowska University, 20-031 Lublin, Poland; marzena.fialek@mail.umcs.pl (M.F.); edyta.wlazlowska@onet.pl (E.W.)

**Keywords:** cerium, multiwalled carbon nanotubes, screen-printed electrode, stripping voltammetry method, application

## Abstract

A simple and fast stripping voltammetric procedure for trace determination of Ce(III) in environmental water samples has been developed. The procedure of cerium determination in the presence of Alizarin S and acetate buffer was employed as the initial method. The adsorption material, multi-walled carbon nanotubes, was used as a screen-printed electrode modifier ensuring efficient accumulation of the Ce(III)-Alizarin S complex. The calibration graph for Ce(III) for an accumulation time of 60 s was linear in the range from 1 × 10^−8^ to 7 × 10^−7^ mol L^−1^ with the linear correlation coefficient r = 0.997. The detection limit was estimated from three times the standard deviation of low Ce(III) concentration and an accumulation time of 60 s was about 3.5 × 10^−9^ mol L^−1^. The proposed method was successfully applied to Ce(III) determination at trace levels in environmental water samples, such as river, lake and rain water with recoveries ranged from 93% to 98%.

## 1. Introduction

Heavy metals are a collection of natural chemical elements that exhibit metallic properties, distinguished by their relatively high density of more than 5 g/cm^3^ [1]. Heavy metals make up the majority of the periodic table of chemical elements, including those present in the scandium group, which includes cerium, the subject of this work. Heavy metals are hazardous to the entire environment. Being present in the air, they also find their way into water or soil, where they are absorbed by living organisms. In them, they accumulate in tissues, causing all sorts of negative health effects, and the increasing concentration of heavy metals in recent years is one of the most troublesome elements of environmental contamination [2,3,4].

Today, almost nothing works without rare earth metals. There would be no smartphones, flat screens, LED lights, batteries, electric motors and many other electronic devices. In high-tech industries such as automotive, electronics, energy or medicine, it is impossible to imagine life without these valuable raw materials. It is therefore of increasing interest to find out where these rare earth elements disappear after use. When analysing wastewater from industrialised areas, it was noted that of the rare earth elements, the highest concentrations were found for cerium. The scientists therefore assume that in the near future elevated concentrations of cerium will also be found in lakes, rivers and groundwater [5,6,7,8,9]. The most popular method for the determination of cerium by far is spectrophotometry, as evidenced by the number of articles available on the spectrophotometric determination of cerium using a range of different reagents to produce coloured reaction products, such as sulphanilic acid [10], N-p-Chlorophenylcinnamohydroxamic acid [11] and Leuco Disulphine Blue [12]. Unfortunately, spectrophotometric methods are not dedicated to field measurements and additionally, in the case of cerium analysis, they often require the use of toxic reagents, but most importantly, the detection limits obtained are unsatisfactory in relation to actual cerium concentrations in environmental waters. Therefore, in our work we proposed the use of stripping voltammetry as a measurement technique for the field determination of trace concentrations of cerium in environmental water samples. The main advantages of voltammetric procedures are, first and foremost, the low cost of the apparatus, the possibility of taking measurements in the field and, very importantly, the possibility of obtaining very low detection limits. Due to its numerous advantages, the stripping voltammetry method is very often used especially for the determination of trace concentrations of many metals [13,14,15,16,17,18].

In typical voltammetric measurements, the measuring system is equipped with three electrodes, working, reference and auxiliary. The reference electrode is a reversible electrode with a fixed potential and when the electrical circuit is closed, the current flows between the working electrode and the auxiliary electrode. A key element in voltammetric procedures is the selection of the working electrode at which the reaction underpinning the quantitative and qualitative analysis takes place. A range of working electrodes are used in the stripping voltammetry method. The type of working electrode used in the determination has, among other things, an influence on the possibility of achieving low detection limits and good separation of analytical signals. The implication of the wide range of electrodes used is that they have an equally wide range of applications [19,20]. Ideally, the working electrode should be made of non-toxic material and allow operation over the widest possible potential range. In the case of field measurements, the working electrode should, in addition, require no additional preparation before measurement and should enable a series of measurements to be made, guaranteeing reproducibility and repeatability. It would be desirable for it to be widely available so that the procedure developed can be applied globally in different laboratories and research groups. These conditions are ideally met by screen-printed electrodes, which take the form of an integrated system consisting of a working electrode, a reference electrode, an auxiliary electrode applied to a non-conductive polymer substrate, and an electrical contact that allows connection to the electroanalyser [13,21,22].

The following voltammetric procedures for the determination of cerium using the following forms of cerium and working electrodes have been described in the literature, Ce(III)-Alizarin S complex at a carbon paste electrode modified with potassium biphthalate [23], Ce(III)-Alizarin S complexon at a carbon paste electrode modified in situ with cetyltrimethylammonium bromide [24], Ce(III)-Alizarin S complex at a glassy carbon electrode [25], Ce(III)-Alizarin S complex at a glassy carbon electrode modified with an antimony film [26], Ce(IIl) at a chemically modified carbon paste electrode containing functionalised nanoporous silica gel [27], Ce(III) at a carbon paste electrode modified by N′-[(2-Hydroxyphenyl)Methylidene]-2-Furohydrazide [28], Ce(IV) at a double-ion imprinted polymer @magnetic nanoparticles modified screen printed carbon electrode [29], Ce(III) at a carbon paste electrode modified with nano-sized cerium-imprinted polymer and multiwalled carbon nanotubes [30], Ce(III) at a carbon paste electrode modified with ion imprinted polymers [31], Ce(III) at a poly(catechol) film modified glassy carbon electrode [32], Ce(III) at an electropolymerized poly-catechol and ion-imprinted membrane modified electrode [33] and Ce(III) at an indium tin oxide electrode [34]. As can be seen, cerium accumulated on the electrode either after the formation of a Ce(III)-Alizarin S complex in the solution or in the form of Ce(III), and as far as the working electrodes were concerned, the vast majority of these were laboratory-modified electrodes. Such modification is a time-consuming process and requires special reagents and special preparation, making such electrodes unsuitable for common field measurements. In our research, we proposed a new procedure based on the accumulation of cerium in the form of Ce(III)-Alizarin S complex on a commercially available screen-printed electrode, which does not require any modification, making our procedure universal and applicable to field measurements and any laboratory. It was crucial to select a suitable material that was used as a working electrode in the screen-printed electrode. It turned out that we achieved very good results by choosing carbon nanotubes which have strong adsorption ability for heavy metals. Thanks to the use of carbon nanotubes as the working electrode material in the screen-printed electrode we used and the selection of a number of parameters, such as the composition and concentration of the basic electrolyte, accumulation potential, and signal recording techniques, we managed to obtain a low detection limit, which makes it possible to use our procedure in field measurements. The basic novelty of our procedure is its universality, thanks to the use of generally available electrodes without the need for additional modification, combined with high sensitivity and the possibility of conducting measurements in the field.

## 2. Results

### 2.1. The Influence of Alizarin S

**Figure 1:**
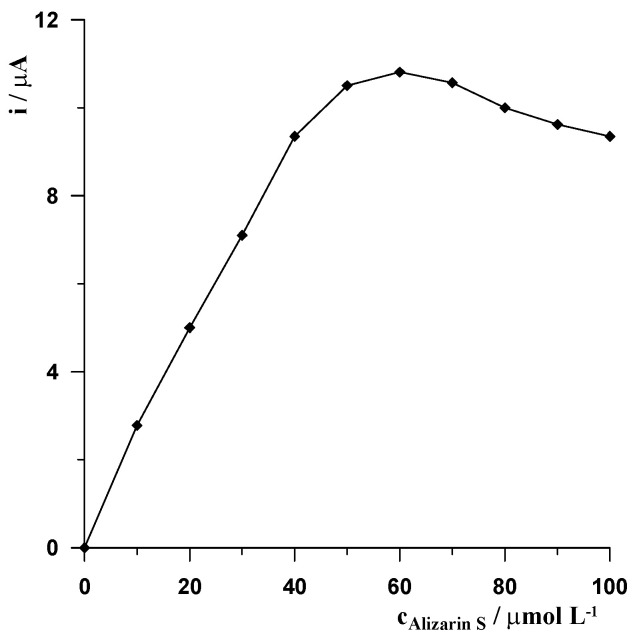
The effect of Alizarin S concentration on the cerium peak current. The pH of the acetate buffer was adjusted to 5.25 ± 0.05, Ce(III) concentration equal to 2 × 10^−7^ mol L^−1^ in all examinations. Accumulation potential −0.3 V and accumulation time 60 s.

The initial measurements carried out without the presence of Alizarin S in the solution showed that when Ce(III) is in an uncomplexed form, it does not concentrate on the MWCNTs/SPCE, which results in a lack of analytical signal. If free Ce(III) ions are to be accumulated on the electrode, as has been demonstrated in many papers, the electrode must be suitably modified, usually using ligands that form complexes with Ce(III). Since the aim of our study was to develop a procedure using commercially available electrodes, without their additional, often complex modifications, to make the procedure widely available for practical use, we proposed that the ligand be added directly to the sample solution to form a complex with Ce(III). As mentioned in the introduction when analysing voltammetric procedures for the determination of Ce(III) described in the literature, when it accumulates from the solution as a complex, Alizarin S is used as the complexone [23,25]. Therefore, in our procedure Alizarin S was also selected for preliminary studies. It was found that with the introduction of Alizarin S into the solution, a cerium signal began to be observed, which proves that the formed Ce(III)-Alizarin S complexes are adsorbed on the unmodified MWCNTs/SPCE. As can be seen in Figure 1, when the concentration of Alizarin S increases to a concentration of 5 × 10^−5^ mol L^−1^, the cerium peak current increases and then, at higher concentrations up to 7 × 10^−5^ mol L^−1^, it remains virtually unchanged and then decreases.

### 2.2. The Influence of pH and Concentration of Acetate Buffer

Analysing voltammetric procedures for the determination of Ce(III) described earlier in the literature and voltammetric procedures for the determination of other metal ions using Alizarin S as a complexing agent, the vast majority are carried out in an acidic environment and acetate buffer is used as the primary electrolyte [23,25]. Therefore, this buffer was initially chosen as the primary electrolyte in our study. The influence of the buffer pH on the Ce(III) voltammetric signal was examined. The tests were conducted in the pH range of 3–6.4. Testing was carried out for a solution containing a Ce(III) concentration of 2 × 10^−7^ mol L^−1^, using the accumulation time and potential of 60 s and −0.3 V, respectively. For each tested acetate buffer, a measurement was made and the current value of the cerium signal was monitored. Analysing the peak currents for different pH values of the acetate buffer, the signal value was found to increase from pH 3.0 to 5.0, a constant signal was obtained up to a pH of 5.5, and then the peak current decreased slightly above pH 5.5. Therefore, an acetate buffer with a pH of 5.25 was chosen as the most optimal buffer, which was prepared to an accuracy of ±0.05 and used in all measurements.

It was noted that in addition to the pH value of the acetate buffer, the concentration of the acetate buffer also influences the value of the cerium peak current. When examining the effect of acetate buffer concentration in the range of 0.075 to 0.125 mol L^−1^, it was observed that the highest cerium peak current was observed for a concentration of 0.1 mol L^−1^, so this was chosen as the optimum.

### 2.3. The Influence of the Potential and Accumulation Time

**Figure 2:**
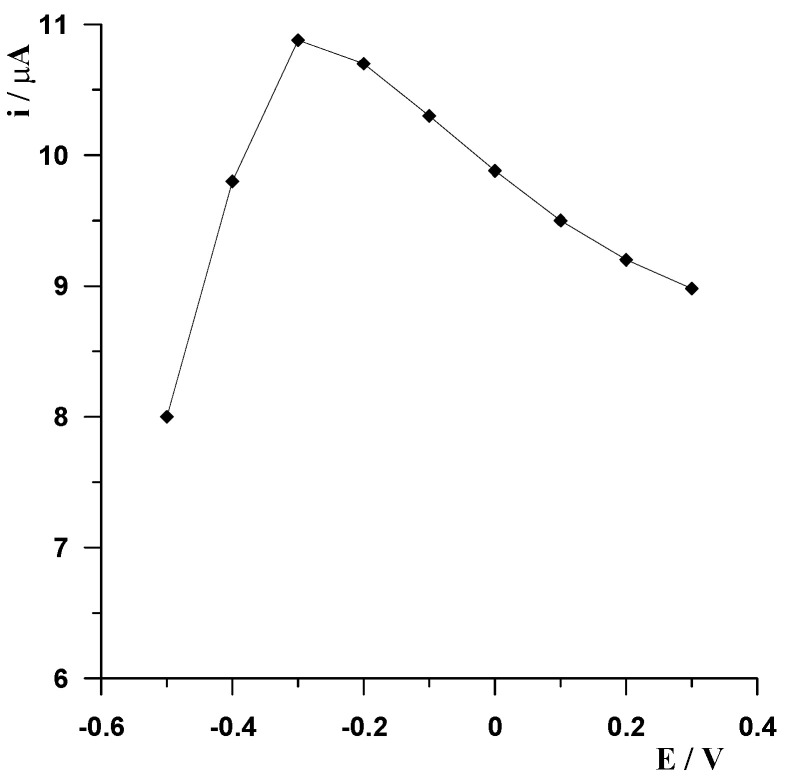
The effect of accumulation potential on the cerium peak current. The pH of the acetate buffer was adjusted to 5.25 ± 0.05, Ce(III) concentration equal to 2 × 10^−7^ mol L^−1^, Alizarin S concentration equal to 5 × 10^−5^ mol L^−1^ in all examinations. Accumulation time 60 s.

The effect of accumulation potential on the stripping peak currents of Ce(III) was studied in the range of −0.5 V to 0.3 V every 0.1 V at standard conditions. The results in Figure 2 show the stripping peak currents as a function of deposition potential. As the accumulation potential became more positive from −0.5 V, the stripping peaks of Ce(III) increased, the maximum peak heights were obtained at a potential of −0.3 V, and then a decrease in peak height was observed at potentials more positive than −0.2 V.

Stripping voltammetry has become a key method in trace analysis of many metals. Thanks to the introduction of the accumulation stage, the sensitivity of determinations can be significantly increased. Also, in the case of our proposed procedure, a significant increase in the cerium peak current value was observed with the increase in the accumulation time. The tests were carried out under standard conditions for solutions containing Ce(III) at concentrations of 1 × 10^−7^ mol L^−1^ and 5 × 10^−7^ mol L^−1^. In both cases, an increase in the peak current value was observed as the accumulation time increased to 60 s.

### 2.4. Repeatability and Reproducibility

The stripping voltammetric measurements were also followed to establish the durability of the sensor. Thus, to examine the repeatability of the MWCNTs/SPCE, six measurement estimations were carried out on 2 × 10^−7^ mol L^−1^ Ce(III) by applying the same electrode in an acetate buffer solution of pH 5.25. Measurements were performed without washing the electrode between measurements. In so doing, the relative standard deviation (% RSD) was found to be 2.8%, justifying the good repeatability of the fabricated material.

In order to study the reproducibility of the designed sensor, three measurement estimations were carried out on 2 × 10^−7^ mol L^−1^ Ce(III) by applying three different electrodes in an acetate buffer solution of pH 5.25. In so doing, the relative standard deviation (% RSD) was found to be 3.5%, justifying the good reproducibility of the fabricated material.

### 2.5. Limit of Detection

**Figure 3:**
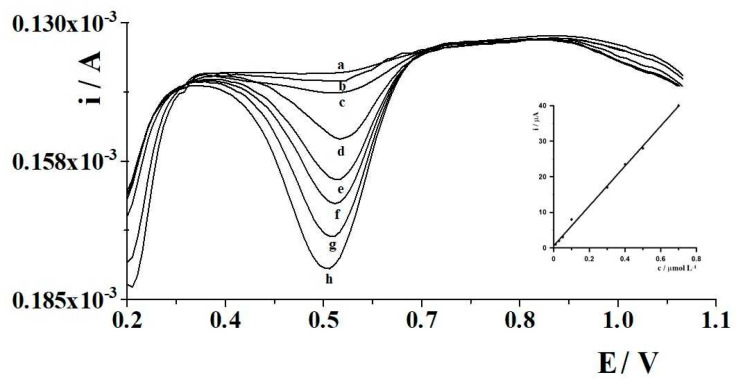
Cerium calibration curve and corresponding voltammograms. The Ce(III) concentrations: 1 × 10^−8^ (a), 3 × 10^−8^ (b), 5 × 10^−8^ (c), 1 × 10^−7^ (d), 3 × 10^−7^ (e), 4 × 10^−7^ (f), 5 × 10^−7^ (g) and 7 × 10^−7^ mol L^−1^ (h).

The calibration graph for Ce(III) for an accumulation time of 60 s was linear in the range from 1 × 10^−8^ to 7 × 10^−7^ mol L^−1^ and obeyed the equation y = 59.08x − 0.55, where y and x are the peak current (µA) and Ce(III) concentration (µmol L^−1^), respectively. The linear correlation coefficient was r = 0.997. Cerium calibration curve and corresponding voltammograms are presented in Figure 3. The detection limit was estimated from three times the standard deviation of low Ce(III) concentration and an accumulation time of 60 s was about 3.5 × 10^−9^ mol L^−1^. As can be seen, the analytical sensitivity of the MWCNTs/SPCE for the determination of Ce(III), compared to the use of a glassy carbon electrode [25], is more than an order of magnitude better, while maintaining excellent repeatability and reproducibility, fast analysis and widespread availability of the sensor.

### 2.6. Selectivity towards Other Metal Ions

**Figure 4:**
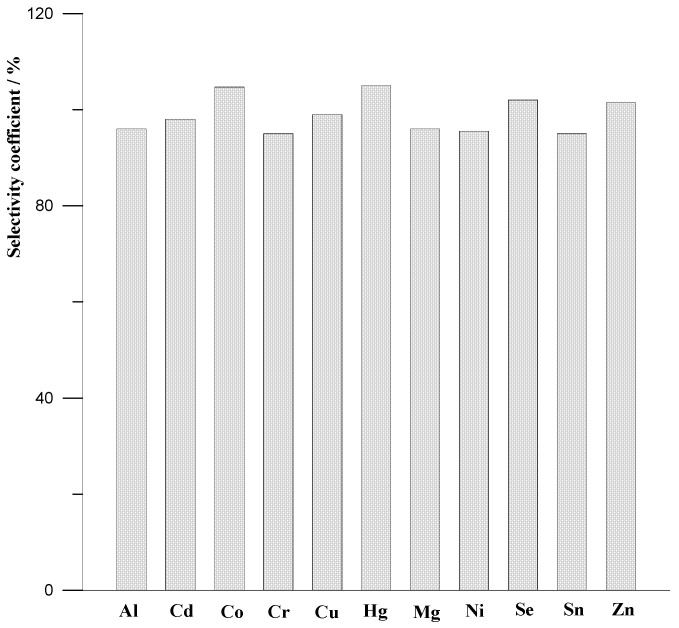
The selectivity coefficients towards other metal ions. The metal ions were taken in a 100:1 ratio with respect to Ce(III).

In addition to the individual estimation of Ce(III), the effect of the intervention of other metal ions on the electroanalytical performance of the MWCNTs/SPCE and its selectivity over cerium was also investigated. By following the optimised procedure, the analysis was performed in the presence of various metal ions, like Al(III), Cd(II), Co(II), Cr(III), Cu(II), Hg(II), Mg(II), Ni(II), Se(IV), Sn(II) and Zn(II), being potentially available in environmental water samples. The metal ions were taken in a 100:1 ratio with respect to Ce(III). The tolerance limit was defined as the concentrations of foreign substances, which gave an error smaller than ±5.0%. The experiments performed proved that the proposed method shows a very good selectivity of Ce(III) towards other ions, without any major external interference. The effect of interfering species on the peak cerium current is shown in Figure 4 as the dependence of the selectivity coefficients determined for each tested metal ion (MI). The selectivity coefficients (SC) were calculated using the following formula: SC = (I_Ce+MI_/I_Ce_) 100%, where I_Ce+MI_ and I_Ce_ are the currents generated for Ce(III) in the presence and absence of co-existing metal ion, respectively.

### 2.7. Study of Interferences

If the application objective of the procedure under development is to be its application to the analysis of environmental samples, it is necessary to investigate how the matrix of such samples affects the signal underlying the qualitative and quantitative analysis. Therefore, in the present study we examined the effects on the cerium signal of both surfactants with different charges and humic substances as indispensable components of environmental waters. Surfactants are of great importance and are used in many industries. However, the large-scale use of surfactants raises risks associated with their entry into the environment, particularly the aquatic environment. Humic compounds are macromolecular compounds with a complex structure, belonging to organic substances. In water, they are formed as a result of the transformation of the organic matter of soils, through the process of humification, i.e., the interaction of physicochemical (such as pH, oxygen, temperature) and fermentation factors (bacteria and fungi) with dead plant remains. Hence, humic substances are a constant component of environmental waters. For our study, we chose one representative from each of anionic (SDS), cationic (CTAB) and non-ionic (Triton X-100) surfactants and a biosurfactant (Rhamnolipids). As humic substances, we used humic acids (HA), fulvic acids (FA) and natural organic matter (NOM) for the study. In order to estimate the insensitivity of our procedure to the components of the environmental water matrix, a laboratory study was carried out by observing the cerium signal in the presence of the above-mentioned substances, in the range of their concentrations from 0.2 to 2 mg L^−1^. It was found that almost all substances in the range tested did not significantly affect the cerium signal, the exception being CTAB, whose presence in the solution at a concentration of 2 mg resulted in a reduction in the cerium peak by about half. Therefore, it can be stated unequivocally that the procedure we developed for the determination of Ce(III) using the MWCNTs/SPCE is resistant to the influence of the matrix of aqueous environmental samples. This is an even more important advantage as in almost all voltammetric procedures for the determination of Ce(III), including those dedicated to the analysis of environmental samples, the effect of surfactants and humic substances has not been studied at all. Only one work has investigated their influence and, comparing the results obtained, it can be concluded that the electrode proposed in our work provides greater resistance and insensitivity to surfactants and humic substances.

### 2.8. Determination in Environmental Waters

**Figure 5:**
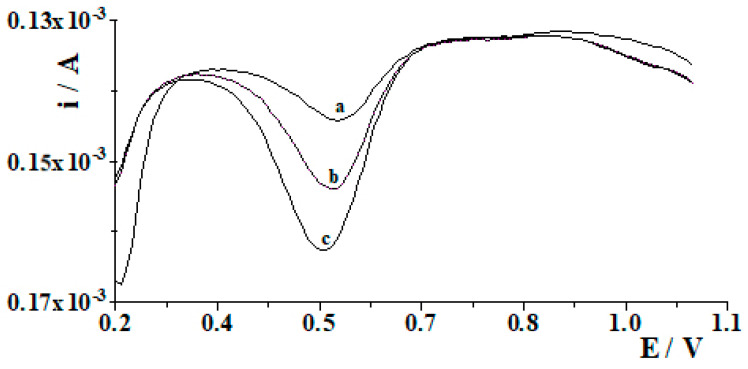
The voltammograms obtained during cerium quantification in Czerniejówka river water diluted (1:10) containing 0.2 µmol L^−1^ Ce(III) (a); as (a) + 2 × 10^−7^ mol L^−1^ Ce(III) (b); as (a) + 4 × 10^−7^ mol L^−1^ Ce(III) (c).

The developed method for the MWCNTs/SPCE used as an electrochemical sensor was further applied to the determination of Ce(III) in environmental water samples. Table 1 shows the results obtained for the detection of cerium ions in river water, lake water and rainwater samples. The water samples were taken for analysis without any prior preparation. The samples were spiked with known amounts of Ce(III) at 5 × 10^−8^ mol L^−1^ level, followed by a voltammetric analysis of these samples to determine the sensitivity and accuracy of the applied MWCNTs/SPCE. Recoveries obtained for the river water, lake water and rainwater samples ranged from 93% to 98%. Example voltammetograms recorded during the analysis of water from the Bystrzyca River are presented in Figure 5. The results demonstrated the applicability of the MWCNTs/SPCE for direct analysis of real samples without any pre-treatment.

## 3. Experimental

### 3.1. Instruments

Electrochemical data were obtained with a three-electrode system using a potentiostat, model Autolab PGSTAT 10 (Utrecht, The Netherlands). A screen-printed carbon electrode (SPCE), model 110CNT manufactured by Metrohm DropSens, was used for the measurements. The cost of one electrode was approximately 6 Euro. This model consists of multi-walled carbon nanotubes (MWCNTs) as the working electrode, carbon as the auxiliary electrode, and silver as the reference electrode. These screen-printed carbon electrodes modified with multi-walled carbon nanotubes (MWCNTs/SPCE) are designed for the development of sensors with an enhanced electrochemical active area and enhanced electronic transfer properties. These electrodes were integrated on a ceramic substrate with the following dimensions: length 33 × width 10 × height 0.5 mm, with silver electric contacts. MWCNTs/SPCEs are commercialised in 50-unit packs. They should be stored at room temperature, in a dry place and protected from light.

### 3.2. Reagents

Ce(III) solutions with a concentration of 0.1 and 0.01 nmol L^−1^ were prepared by dilution of 1 g L^−1^ of highest purity stock standard solution (Merck, Hong Kong, China). A solution of 1 × 10^−2^ mol L^−1^ Alizarin S (sodium alizarin sulfonate) was prepared by dissolving 0.1712 g of the reagent in water in a 100 mL flask and stored in a refrigerator at a temperature of 6 ± 1 °C. The solutions of 1 mol L^−1^ of the acetate buffers were prepared from acetic acid and sodium hydroxide (Suprapur, Merck). Standard solutions of surface active substances: Triton X-100, cetyltrimethylammonium bromide (CTAB), sodium dodecyl sulfate (SDS) and rhamnolipid (biosurfactant), were purchased from Fluka (Buchs, Switzerland). Humic acid sodium salt (HA) was obtained from Aldrich. River fulvic acid (FA) and natural organic material (NOM) were obtained from the Suwannee River and purchased from the International Humic Substances Society. The water used for all analysis was deionised in a laboratory purification Milli-Q system. All other reagents used were of analytical reagent grade or Suprapur.

### 3.3. Procedure

In order to determine Ce(III) in an aqueous sample, the screen-printed carbon electrode (SPCE) modified with multi-walled carbon nanotubes (MWCNTs) was inserted into the analysed solution reached with acetate buffer pH = 5.3 and Alizarin S. The final volume was 10 mL and the acetate buffer and Alizarin S concentrations were 0.1 mol L^−1^ and 5 × 10^−5^ mol L^−1^, respectively. For voltammetric measurements, the following parameters were chosen: before each measurement, the electrode was cleaned using the electrochemical method by applying a potential of 1.1 V to it for 10 s, then after an accumulation step carried out for 60 s at −0.3 V, the differential pulse voltammogram was recorded, while the potential changed towards positive values up to 1.1 V. In this potential range, Ce(III) was oxidised to Cr(IV) and the peak current value corresponding to this process was visible at a potential value of 0.5 V. The scan rate was 0.0795 V/s.

**Figure 6:**
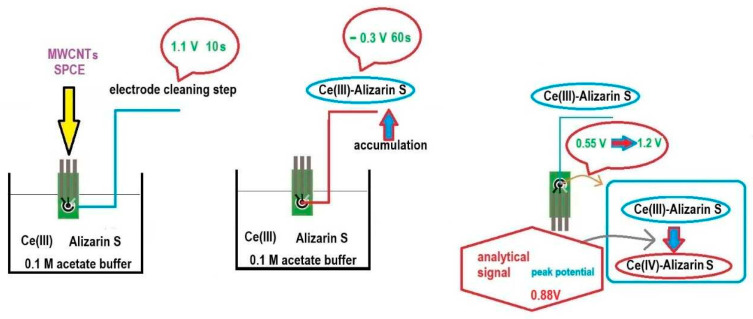
Schematic diagram showing the voltammetric measurement.

The procedure is graphically illustrated in Figure 6.

## 4. Conclusions

In comparison with the procedures for Ce(III) determination described in the literature, the present work describes a very simple, sensitive, selective and low-cost method that can monitor concentrations of cerium ions in environmental water samples. This was achieved by using carbon nanotubes as the adsorption material in the screen-printed electrode, on which the Ce(III)-Alizarin S complex was accumulated. The procedures described earlier in the literature required, for the most part, time-consuming preparation of the electrodes on which cerium concentration occurred. Importantly, these procedures are not suitable for widespread use due to the lack of common availability of these electrodes. The electrode proposed in our procedure is widely available and the entire measurement procedure has been designed to make analysis simple and feasible in any laboratory or in the field and the total voltammetric measurement time, including electrochemical cleaning of the electrode, takes just over one minute. The influence of the matrix of environmental samples was investigated in detail and, as proven, the measurements are undisturbed in the presence of a wide range of both surfactants and humic substances. This is a further advantage because in almost all voltammetric procedures for the determination of cerium, the influence of these substances on the analytical signal obtained was not investigated. Our proposed procedure is also characterised by very good selectivity of Ce(III) towards commonly occurring other metal ions. The insensitivity of our procedure to the matrix of environmental water samples and the good selectivity were associated with the perspective of its practical application in environmental water monitoring.

## Figures and Tables

**Table 1:** Analytical results of Ce(III) determination in natural water samples by the proposed standard procedurę. Ce(III) found was calculated using the standard addition method.

Sample	Ce(III) Added (µmol L^−1^)	Ce(III) Found (µmol L^−1^)	Recovery (%)	RSD (*n* = 5) (%)
Czerniejowka river water	0.100	0.094	94.0	6.2
	0.200	0.191	95.5	4.9
Lake Zemborzyce	0.100	0.093	93.0	5.8
	0.200	0.193	96.5	5.3
Rain Water	0.100	0.096	96.0	4.4
	0.200	0.196	98.0	4.7

## Data Availability

The data presented in this study are available in article.
